# Progress in Surgery of the Stomach

**Published:** 1903-10-03

**Authors:** 


### Progress in Surgery of the Stomach.

**The Surgical Treatment of Gastric and Duodenal Ulcers** is discussed by Moynihan.[1] Perforation may be acute, subacute or chronic: acute when the ulcer gives way suddenly and completely and the stomach contents are free to escape at once into the general peritoneal cavity; subacute when the ulcer perforates rapidly but the stomach is empty, the opening small or quickly closed by adhesions or a plug of omentum; and chronic, when owing to limiting adhesions to the anterior abdominal wall, the liver, the pancreas, or any neighbouring structure, the escape of contents is slight and confined by barriers of lymph to a circumscribed area, with the result that an abscess slowly forms and becomes manifest at a later period. In acute and subacute perforation an operation should be advised as soon as the diagnosis is assured. Taking all cases into account, and including those in which death results, perhaps remotely from subphrenic or perigastric abscess, he estimates the average recoveries at 35 to 40 per cent. The rent is sewn up by a double layer of continuous suture without excising the ulcer, and he generally flushes the peritoneum if much soiled with hot saline solution; if little soiled he wipes it clean with large swabs dipped in saline solution and at once used without being squeezed. Drainage is very rarely necessary. The characteristics of hæmorrhage (which is often the first symptom) from an acute ulcer he describes as spontaniety, abruptness of onset, the rapid loss of a large quantity of blood, the marked tendency to spontaneous cessation, and the infrequency of a repetition of hæmorrhage in anything but insignificant quantity. In a majority of such cases the blood has been observed to come from many simple erosions on the mucous membrane. The bleeding from a chronic ulcer varies within the widest limits of frequency and quantity, but roughly speaking we may say that the cases are divisible into two groups. In the one, the hæmorrhage is trivial in amount, capricious in onset, and irregularly repeated and is merely an unexpected and, on the whole, an unimportant addition to the usual attacks of vomiting. In the second group, the hæmorrhage is the predominant feature, it is copious and tends to return. The treatment of these cases of hæmatemesis depends upon the nature of the ulcer from which the blood is coming. In hæmorrhage from an acute ulcer medical treatment alone will suffice; surgical measures will rarely be necessary. If any operation has to be done, gastro-enterostomy will probably prove to be the most effective. In chronic ulcer operation should be advised as early as possible. If readily exposed and not adherent to the pancreas or other organ, the ulcer if solitary may be excised, but a simple gastro-enterostomy is probably sufficient to secure the arrest of the hæmorrhage and the rapid healing of the ulcer. For cases of chronic ulcer producing stenosis, dilatation or inveterate dyspepsia, posterior gastro-enterostomy is the operation of choice. Of 18 cases seen by Mansell Moullin,[2] in five no operation was performed, and they all died. In the 13 others an operation was performed; in nine for hæmatemesis, in four for persistent pain and vomiting; and of these 13 two died. Hæmorrhage into the stomach is an indication for operation when it is either excessive or long continued. If the first hæmorrhage of an acute ulcer is only moderate in amount, so that the patient rallies well, but is followed by another attack within a few days, in spite of absolute rest, the bleeding point should be exposed and dealt with on ordinary surgical principles without delay. The same argument holds good for those cases in which hæmatemesis returns after long intervals of apparently perfect health. This is the condition which he has described as recurrent ulceration of the stomach, and which is often mistaken for the chronic form. Whatever may be the cause, acute ulcers form again and again, each with the risk of hæmatemesis and perforation. In two cases of gastric hæmorrhage Max Einhorn[3] has injected twice daily about 1 gram of 1—1,000 solution of adrenalin with apparently good result. When surgical measures are demanded because of profuse gastric or duodenal hæmorrhage, the bleeding point should be sought for and cauterised; but if it cannot be readily located it may be necessary to do a gastro-enterostomy. Cases of operation for gastric ulcer are recorded by Buck,[4] Fraser,[5] Groves,[6] and Jones[7]. Vianay[8] points out that the occasional occurrence of an adherent colon between the upper surface of the liver and the diaphragm annuls the value of absence of liver dulness as a sign of perforation of the stomach or intestine.

**Cancer of the Stomach.**—Robson[9] speaks of the desirability of early diagnosis in these cases. The three most constant symptoms are pain, vomiting, and tumour. In 86·6 per cent. pain is present, in 85·3 per cent. vomiting is present, and in 76·6 per cent. a tumour is present. The nearer the pylorus the more severe the pain. While free hydrochloric acid in good quantity is against a diagnosis of cancer, the absence of free hydrochloric acid is decidedly in favour of it, as in 80 to 90 per cent. of cases of cancer of the stomach free hydrochloric acid is absent, or if present it is in very small quantity. An excess of free hydrochloric acid is decidedly in favour of ulcer, just as the presence of lactic acid is decidedly in favour of cancer. A microscopic examination of the stomach contents may show portions of growth and cancer cells which are pathognomonic. The Oppier-Boas bacillus is said to be present in 90 per cent. of all cases of gastric cancer. Touche states that there is a notable diminution in the number of red corpuscles present in the blood, but they rarely fall below 1,500,000 per cubic millimetre. Robson considers that he advances sufficient evidence to prove:—1. How desirable it is to make an early diagnosis of cancer of the stomach in order that a radical operation may be performed at the earliest possible moment. 2. That it may be needful to perform an exploratory operation in order to complete or confirm the diagnosis. 3. That such an exploration may be done with little or no risk in the early stages of the disease. (4) That even when the disease is more advanced and a tumour perceptible, an exploratory operation is as a rule still advisable in order to carry out radical or palliative treatment. (5) That where the disease is too extensive for any radical operation to be done, the palliative operation of gastro-enterostomy, which can be done with very small risk, may considerably prolong life and make the remainder of it much more comfortable and happy. (6) That some cases, thought at the time to be cancer, too extensive for removal, may after gastro-enterostomy clear up completely and get quite well. (7) That in cases of disease of the cardiac end of the stomach too extensive for removal, the operation of gastrostomy may considerably prolong life and prove of great comfort to the patient by preventing death from starvation. (8) That even where the disease is too extensive either for removal or a gastro-enterostomy being performed with a fair chance of success, the operation of jejunostomy may occasionally prove of service to the patient. (9) That where a radical operation can be performed the thorough removal of the disease may bring about as much relief to the patient as does the operation for the removal of cancer in other organs of the body, and that in some cases a complete cure may follow. Moynihan [10] describes a case of partial gastrectomy for cancer, and forms the following conclusions on this subject: 1. That malignant disease of the stomach begins in the majority of instances near the pylorus just below the lesser curvature. 2. That from this point it spreads most rapidly and most widely in the mucosa. 3. That the rate of growth towards the cardiac orifice is rapid, towards the duodenal side extremely slow. The duodenum is rarely affected extensively. 4. That the tendency of the growth is to drift towards the curvatures. The lymphatic system of the stomach is comparatively simple. There are three chief lymphatic areas: 1. An area along the lesser curvature from which the lymphatic vessels pass upwards and to the left into the coronary glands. The coronary glands lie along the artery of the same name. At the cœliac axis they become continuous with the glands along the upper border of the pancreas. 2. An area along the greater curvature from which the lymphatic vessels pass downwards and to the right into the glands lying along the greater curvature. These glands are more numerous near the pylorus, and from here pass to the head of the pancreas and become continuous with the hepatic group of glands which lie along the hepatic artery, and in part along the pyloric artery. 3. In addition to these two areas is a third, for which he suggests the name "isolated area." This area comprises the greater tuberosity of the stomach, the lower end of the œsophagus, and an area along the greater curvature as far, approximately, as the limit of supply of the gastro-epiploic artery. The lymphatic vessels pass downwards to the hilum of the spleen. This region is very rarely affected by growth spreading upwards from the pylorus. It remains unaffected to the last, and its lymphatic vessels and glands are very rarely involved in the spread of the disease. An operation, therefore, which aims at eradicating the primary growth, the lymphatic vessels draining the area in which the primary growth occurs, and the lymphatic glands into which these vessels drain will involve a removal of all the stomach except the "isolated area" and of the glands along the lesser and greater curvatures. Thomas [11] records a case in which more than three quarters of the stomach were successfully removed for cancer.

[1] Lancet, Jan. 31, 1903, p. 294, and Brit. Med. Jour., Jan. 31, 1903, p. 253. [2] Clin. Jour., Nov. 19, 1903, p. 72, and Brit. Med. Jour., April 25, 1903, p. 953. [3] Med. Rec., New York, March 7, 1903, p. 396. [4] Lancet, April 4, 1903, p. 960. [5] Brit. Med. Jour., Feb. 21, 1903, p. 427. [6] Lancet, Dec. 13, 1902, p. 1621. [7] Brit. Med. Jour., Nov. 29, 1902, p. 1701. [8] Lancet, June 6, 1903, p. 1608. [9] Brit. Med. Jour., April 25, 1903, p. 949. [10] Ibid., p. 955. [11] Lancet, May 30, 1903, p. 1519.

(*To be continued.*)

